# Molecular epidemiology of multidrug resistant extended spectrum beta-lactamase producing *Klebsiella pneumoniae *at a Jamaican hospital, 2000 - 2004

**DOI:** 10.1186/1471-2180-10-27

**Published:** 2010-01-28

**Authors:** Nicole A Christian, Karen Roye-Green, Monica Smikle

**Affiliations:** 1 Department of Microbiology, University of the West Indies, Mona, Kingston, Jamaica

## Abstract

**Background:**

The accurate identification of a pathogen beyond the species level is critical in epidemiological studies and investigations of nosocomial outbreaks of infection. The clonal relatedness of 66 multidrug resistant (MDR) strains of extended spectrum beta-lactamase (ESBL) producing *K. pneumoniae *isolated from clinical specimens from hospitalized patients at a Jamaican hospital during a 5 year period were determined by pulsed field gel electrophoresis (PFGE).

**Results:**

A total 10 different ESBL producing *K. pneumoniae *genotypes designated Clones I-X were found. The most frequently occurring strains belonged to Clones I (21/66, 32%), II (15/66, 26%), III (13/66, 20%) and IV (8/66, 12%) which accounted for 86% (57/66) of ESBL producing *K. pneumoniae *strains over the 5 year period. The remaining 9 (14%) cases of ESBL producing *K. pneumoniae *were due to strains of Clones V-X. The 4 predominant clones persisted for several years in the hospital.

**Conclusions:**

The clonal and temporal distribution of the MDR ESBL producing *K. pneumoniae *strains among clinical service areas did not suggest outbreaks of the organism during the period of study. Instead the molecular epidemiology of ESBL producing *K. pneumoniae *at this hospital was more representative of an endemic persistence of clones of the organism with limited dissemination from patient to patient. Further studies to investigate the factors which determine the emergence and persistence of MDR ESBL producing *K. pneumoniae *in Jamaican hospitals and their impact on clinical and economic outcomes at such institutions would be useful.

## Background

The increasing prevalence of multidrug resistant (MDR) pathogens causing nosocomial infection constitutes a major health problem [1]. *Klebsiella pneumoniae *ranks among the top ten organisms causing blood stream infection, pneumonia and other invasive infections in hospitalized patients in different countries [2-4]. An increasing prevalence of multidrug resistant strains of *K. pneumoniae *which possess extended spectrum beta-lactamases (ESBL) enzymes, encoded by plasmid-borne genes which confer resistance to broad spectrum cephalosporins and other antibiotics used to treat serious infection has been widely reported [2]. Multidrug resistance contributes to unfavourable clinical outcomes, impacts the utilization of hospital resources, increases the burden of effective infection control practice and the overall health economic cost [1,2].

The prevalence of ESBL producing strains of *K. pneumoniae *differs between countries. In the developing world a recent study from Jamaica reported that almost one-fifth of *K. pneumoniae *isolates at a tertiary referral teaching hospital were ESBL producers [5]. The presence of ESBL-producing Gram negative bacilli in hospitals in other Caribbean islands also has been reported [6,7].

This study reports the clonal relationships of MDR ESBL producing *K. pneumoniae *at a Jamaican hospital.

## Results

The majority of the MDR *K. pneumoniae *isolates were from urine specimens (31/66, 47%), blood (9/66, 13%) and sputum (7/66, 10%). Almost a third (19/66, 29%) were isolated from children admitted to paediatric wards while 15% (10/66) were from intensive care unit (ICU) patients. The remaining 37 strains were isolated from patients admitted to medical (n = 15), surgical (n = 9), special care nursery (n = 5), orthopaedic (n = 5) and obstetrics/gynaecological services (n = 3).

As shown in Table 1, in addition to ceftazidime, the majority of the isolates were resistant to trimethoprim/sulfamethoxazole (59/66, 89%) and the aminoglycosides (tobramycin 50/66, 76% and gentamicin 49/66, 74%). All (66/66, 100%) isolates were susceptible to meropenem.

**Table 1:** Antibiotic susceptibilities of 66 strains of multidrug resistant (MDR) extended spectrum beta - lactamase (ESBL) producing *K. pneumoniae*, 2000-2004

Antibiotic	Susceptibility (%)
Nalidixic Acid	82

Norfloxacin	88

Ciprofloxacin	91

Levofloxacin	85

Gentamicin	26

Tobramycin	24

Minocycline	59

Nitrofurantoin	9

Trimethoprim/sulfamethoxazole	11

Ceftazidime	0

Cefepime	0

Meropenem	100

All 66 (100%) isolates of MDR *K. pneumoniae *tested positive for ESBL production in the double- disc synergy test and the E-Test ESBL screen. The E-test ESBL screen showed that all isolates (66/66; 100%) had MIC ceftazidime and cefepime > 32 μg/ml and > 16 μg/ml, respectively. The MICs were subsequently determined by the agar gel dilution method which revealed MICs ranging from 32 - >1024 μg/ml for ceftazidime and 2 - >1024 μg/ml for cefepime indicating ESBL production by all (66/66; 100%) strains.

**Figure 1:**
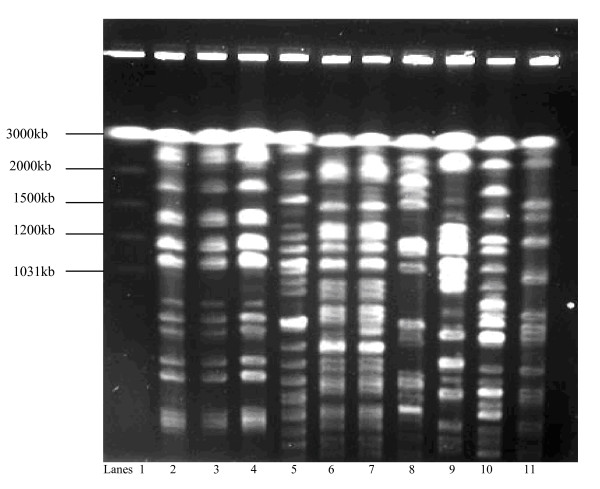
**Pulsed field gel electrophoresis (PFGE) analysis of XbaI digests of multidrug resistant (MDR) *K. pneumoniae *strains from intensive care unit (ICU) patients (2000-2004)**. Lane 1: molecular size marker, *Saccharomyces cerevisiae*; lanes 2-4: MDR *K. pneumoniae *Clone I isolated during 2001; lane 5: Clone II isolated during 2002; lanes 6-7: *K. pneumoniae *strains belonging to Clones III, isolated 2 weeks apart from the same patient; lanes 8-9: Clones V and VI isolated in 2003; lanes 10-11: Clones VII and VIII, respectively isolated from the same patient during 2003.

**Figure 2:**
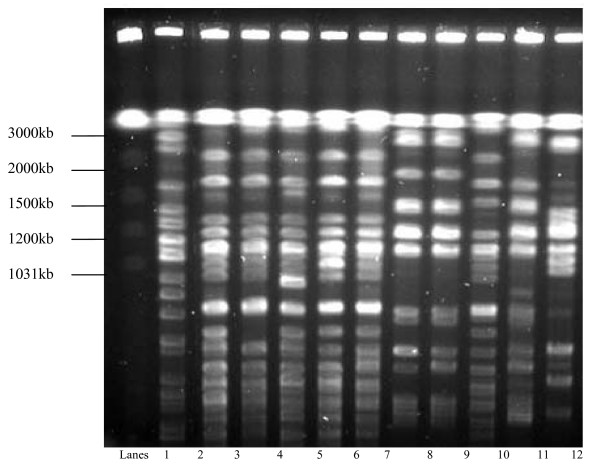
**Pulsed field electrophoresis (PFGE) analysis of XbaI digests of multidrug resistant (MDR) *K. pneumoniae *strains isolated from paediatric patients (2000-2004)**. Lane 1: molecular size marker, *Saccharomyces cerevisiae*; lane 2: *K. pneumoniae *Clone III isolated during 2001; lanes 3-7: five strains of *K. pneumoniae *Clone II isolated from specimens collected from the same patient during the same day; lanes 8-9: Clone I isolated from unrelated patients during 2002; lane 10: Clone II isolated during 2002; lane 11: Clone I isolated during 2003 and lane 12: Clone VI isolated during 2004.

**Figure 3:**
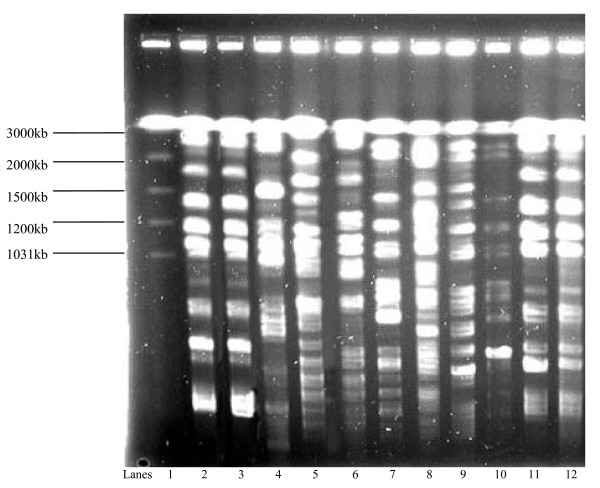
**Pulsed field electrophoresis (PFGE) analysis of XbaI digests of 11 multidrug resistant (MDR) *K. pneumoniae *strains isolated from patients admitted to the paediatric wards (2000-2004)**. Lane 1: molecular size marker, *Saccharomyces cerevisiae*; lanes 2-3: two strains of MDR *K. pneumoniae *clone I isolated from the same patient during 2001 and 2002, respectively; lane 4: MDR *K. pneumoniae *clone III isolated during 2001; lanes 5-6: clone II; lanes 7-8: clones IV and III from the same patient during the same admission in 2002; lanes 9-10: clone IV; and lanes 11-12: clone I strains from different patients.

**Figure 4:**
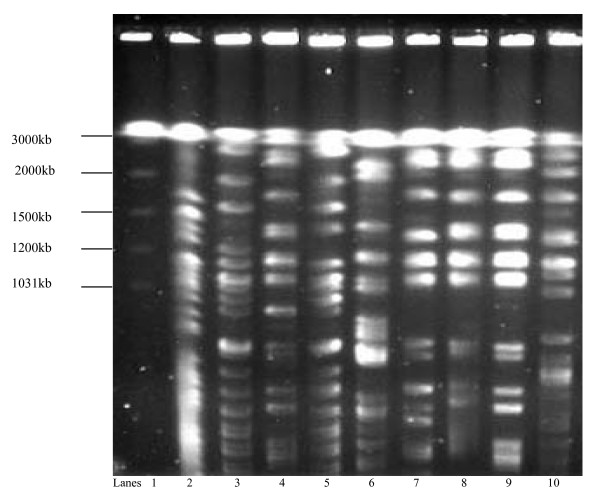
**Pulsed field electrophoresis (PFGE) analysis of XbaI digests of 9 multidrug resistant (MDR) *K. pneumoniae *strains (2000-2004)**. Isolates were obtained from patients admitted to the orthopaedic ward (lanes 2-6) showing PFGE patterns corresponding to clone IX (lane 2), clone II (lanes 3 and 5), clone I (lane 4) and clone IV (lane 6), 2000-2002; and the medical wards (lanes 7-10) showing PFGE patterns of clone I (lanes 7-9) and clone II (lane 10), 2002-2003.

The PFGE of XbaI digests of chromosomal DNA from the 66 ESBL producing *K. pneumoniae *strains revealed 10 banding patterns representing 10 genotypes which were designated Clones I-X. The most frequently occurring were Clones I (21/66, 32%), II (15/66, 23%), III (13/66, 20%) and IV (8/66, 12%). Multiple genotypes in comparable frequencies were isolated from specimens from various clinical service areas. The PFGE analysis of the MDR *K. pneumoniae *from patients admitted to different clinical service areas and the banding patterns are shown in Figures 1, 2, 3 and 4. There were 8 cases of MDR *K. pneumoniae *infection in long stay patients at the hospital. Among these, coinfections with multiple genotypes of MDR *K. pneumoniae *were observed in 2 admissions in ICU and Paediatrics as shown in Figure 1 (lanes 10 and 11) and Figure 3 (lanes 7 and 8), respectively. Repeat infections occurred in 2 re-admissions after 3 months and 18 months. In the first case, a different clone was involved while in the other the same clone was identified (shown in Figure 3 lanes 2 and 3).

The temporal distribution of the ESBL producing *K. pneumoniae *clones among various hospital services over the 5 year period is summarized in Table 2. There were 7 ESBL producing *K. pneumoniae *isolates during 2000, 12 during 2001, 30 during 2002 and 12 and 5 isolates during 2003 and 2004, respectively. The MDR ESBL *K. pneumoniae *strains belonging to Clones I, II, III and IX were isolated from patients in 4 different clinical service areas during 2000. Clones I and II were first identified in infants on the paediatric wards during July and August and Clone I in 2 patients on the medical wards during September of that year. Clones I-IV were present in the hospital during 2001 with multiple genotypes occurring in 3 of the 6 clinical service areas. The increased prevalence of ESBL producing *K. pneumoniae *observed in the hospital during 2002 involved strains belonging to Clones I-IV. However all 7 clinical service areas were affected but no new genotypes were identified in that year. In contrast the subsequent decline in the frequency of isolates during 2003 was accompanied by the emergence of new genotypes including Clones V-VIII which were identified in clinical specimens from 3 ICU patients and the reemergence of clone I in the hospital after an absence of 10 months. During 2004 3 of 5 isolates from patients admitted to Surgery and Paediatrics belonged to Clone VI.

**Table 2:** Temporal distribution of multidrug resistant (MDR) extended spectrum beta-lactamase (ESBL) producing *K. pneumoniae *clones by hospital service during 2000-2004.

Year	Number of Isolates	Clone/genotypes identified	Hospital Service
2000	7	I, II, III, IX	Paediatrics, Medicine, Orthopaedics, Obstetrics & Gynaecology

2001	12	I, II, III, IV	Intensive care unit, Paediatrics, Surgery, Special Care Nursery, Orthopaedics, Obstetrics & Gynaecology

2002	30	I, II, III, IV	Intensive care unit, Paediatrics, Medicine, Surgery, Special Care Nursery, Orthopaedics

2003	12	I, II, III, IV, V, VI, VII, VIII, X	Intensive care unit, Paediatrics, Medicine, Surgery, Special Care Nursery

2004	5	III, IV, VI	Paediatrics, Surgery

As shown in Table 3, based on the antibiotic susceptibility testing 13 antibiotypes (R1-R13) were identified. There were 22 (33%) quinolone-resistant isolates which were assigned antibiotypes R1-R7. The isolates assigned antibiotype R1 were resistant to all the quinolones tested. The remaining 44 isolates were quinolone sensitive and were assigned antibiotypes R8-R13. No correlations were found between the antibiotypes and genotypic clones of the MDR ESBL producing *K. pneumoniae*. The strains which had similar antibiotypes often belonged to different PFGE clones. However, all 6 isolates with quinolone-sensitive antibiotypes R9 and R13 belonged to PFGE Clone 1 as shown in Table 3.

**Table 3:** The antibiotypes and pulsed field gel electrophoresis (PFGE) clones of the 66 multidrug resistant (MDR) extended spectrum beta-lactamase producing (ESBL) *K. pneumoniae *strains, 2000-2004

Antibiotypes (n)*	Resistance Profile †	*Clones of ESBL K. pneumoniae*
R1 (9)	NA, Nor, Cip, Lev, Cn, Tob, Min, F, SXT	*I, II, III, VIII*

R2 (1)	NA, Nor, Cip, Lev, Cn, Tob, Min, SXT	*VI*

R3 (3)	NA, Nor, Cip, Lev, Cn, Tob, SXT	*III, VII*

R4 (3)	Lev, Cn, Tob, Min, F, SXT	*I, II, IV*

R5 (5)	NA, Cn, Tob, F, SXT	*I, II*

R6 (1)	NA, Cn, Tob, SXT	*II*

R7 (1)	Lev, F	*I*

R8 (2)	Min, Cn	*I, II*

R9 (3)	F	*I*

R10 (6)	SXT	*I, II, III, IV, VI*

R11 (15)	Tob, SXT	*I, II, III, IV, VI*

R12 (14)	Cn, Tob, F, SXT	*I, III, IV, IX, X*

R13 (3)	Cn, Tob, Min, F, SXT	*I*

* n is the total number of MDR *K. pneumoniae *assigned to each antibiotype

† NA nalidixic acid, Nor norfloxacin, Cip ciprofloxacin, Lev levofloxacin, Cn gentamicin, Tob tobramycin, Min minocycline, F nitrofurantoin, SXT trimethoprim sulfamethoxazole

## Discussion

The clonal and temporal distributions of the MDR ESBL producing *K. pneumoniae *strains among clinical service areas in the hospital do not suggest outbreaks of the organism at that institution during the period studied. Instead the epidemiology of ESBL producing *K. pneumoniae *at this hospital is more representative of an endemic persistence of clones of the organism with limited dissemination from patient to patient. However, the persistence of related clones over the time period suggests patient to patient transmission or healthcare worker to patient transmission. The emergence and reemergence of Clone I in the ICU during a 6-month period during 2001 is consistent with this concept. Also the results suggest that there has been transmission of some common strains including PFGE Clones I, II and III, over time in the hospital during 2000-2004. This might have been more evident if asymptomatic patients had been screened for MDR *K. pneumoniae *colonization. The presence of asymptomatically colonized patients may explain the intermittent appearances of certain strains over time in various hospital services. The epidemiology of ESBL producing *K. pneumoniae *at this hospital proved complex and, as explained by Branger et al [8], may involve the spread of self-transferable plasmids as well as clonal spread [8].

Studies conducted in hospitals elsewhere have reported the spread of single clones of MDR *K. pneumoniae *among patients hospitalized over protracted periods of time [8,9]. In the present study ESBL producing *K. pneumoniae *strains belonging to Clone III persisted in the hospital over the 5-year period studied. During 2002 the year in which the largest number of cases, especially of paediatric cases, was seen different genotypes of the organism coexisted in patients on the same wards. This makes it less clear whether or not outbreaks caused by single different strains or involving the 4 endemic clones in the hospital had occurred. The prevalence of ESBL producers at the University Hospital of the West Indies for that year was 18% [5]. The factors contributing to the increasing incidence of ESBL producing *K. pneumoniae *during 2002 have not been clearly defined at this hospital [5]. A number of risk factors for increased colonization with MDR *K. pneumoniae *including the use of third generation cephalosporins have been reviewed [10].

Other interesting observations from the study include the cases of long stay and repeat patients who remained colonized or had repeat infections with the same genotype after long periods of time and those with concomitant infections with different genotypes of ESBL producing *K. pneumoniae*. Branger et al [8] reported the case of a patient colonized with the same ESBL producing *K. pneumoniae *strain for 10 months [8]. Sequential or simultaneous isolation of unrelated strains of ESBL producing *K. pneumoniae *from individual patients has been reported by others [11]. Weller et al [12] reported that multiple subvariants of a strain could persist in an infective population without any one subvariant becoming dominant [11,12].

The previously reported decreased susceptibility to aminoglycosides, fluoroquinolones and trimethoprim/sulfamethoxazole in ESBL producing *K. pneumoniae *was also observed in this study [13]. The data on antibiotypes provided additional evidence in support of the clonality of the PFGE genotypes. The predominant ESBL producing *K. pneumoniae *genotypes I, II, III and IV had the quinolone-resistant antibiotype R1. This might have contributed to the endemic persistence of these clones in the hospital [14]. The more susceptible clones were probably not endemic strains at the UHWI and may have been harboured by patients who were already colonized on admission [8]. Notably, the PFGE genotypes V, VII and VIII isolated from ICU patients also had the more resistant antibiotype R1 though found in lower numbers. A number of factors including aggressive antibiotic therapy, prolonged hospitalization and the performance of invasive procedures are well documented contributors to the increased risk of infection with nosocomial strains of MDR *K. pneumoniae *in patients admitted to the ICU [15].

Clearly different antibiotic susceptibility patterns distinguish different strains of ESBL producing *K. pneumoniae *as shown in the current study. However, antibiotic susceptibility testing has relatively limited utility as a typing system in epidemiologic studies not only because of phenotypic variation but also because antibiotic resistance is under extraordinary selective pressure in contemporary hospitals [14]. The selective pressure from antimicrobial therapy may alter the antimicrobial susceptibility profile of an organism, such that related organisms show different resistance profiles [16]. Graffunder et al [10] found a correlation between the selective pressure of antimicrobial agents identified as risk factors for ESBL producing organisms and the presence of related resistance genes residing on the plasmids [10]. Woodford et al [16] also suggests that antibiotic pressure may have been a factor for initial colonization of patients and the development of further resistance by the organism [16].

The limitations of the study are those attending studies involving retrospective data collection, the disproportionately small number of ESBL producing *K. pneumoniae *strains from some clinical service areas, the long time period over which the isolates were collected, the lack of surveillance cultures to detect asymptomatic, colonized patients with MDR ESBL producing *K. pneumoniae *and the limited available epidemiologic data to compare with the PFGE typing results. During the extended period of study advances in medical technology, changes in patient population, formulary restrictions and changes in standards of practice or infection control measures may affect the results [10].

## Conclusions

In summary the results showed clonal diversity of MDR ESBL producing *K. pneumoniae*, elements of its temporal distribution which were suggestive of endemic persistence and dissemination of this organism between patients at this hospital, the extent of which was not fully ascertained. Further studies which investigate the factors which determine the emergence and persistence of ESBL producing *K. pneumoniae *in Jamaican hospitals and the impact on clinical and economic outcomes at such institutions would be useful.

## Methods

### Microbiological Investigations

All clinical isolates (n = 66) of MDR *K. pneumoniae *from clinical specimens collected from 57 patients admitted to the University Hospital of the West Indies (UHWI), Kingston, Jamaica, a tertiary care referral centre, between June 2000 and April 2004 were stored in tryptose soy broth at -70°C until required. Standard microbiological procedures were followed for the different clinical specimens [17]. Bacterial isolates were identified and the initial antibiotic susceptibility testing was done using the Vitek automated system (Biomerieux, Durham, North Carolina, U.S.A.). The appropriate antibiotic panel for each type of specimen was used as recommended by the manufacturer. The breakpoints for antibiotic susceptibility were determined according to the guidelines of the Clinical and Laboratory Standards Institute (CLSI) [17]. The antibiotics tested included amoxicillin/clavulanic acid, ampicillin, carbenicillin, cefazolin, ceftriaxone, cefuroxime, cephalothin, ceftazidime, ciprofloxacin, gentamicin, levofloxacin, minocycline, nalidixic acid, nitrofurantoin, norfloxacin, ticarcillin/clavulanic acid, tobramycin, trimethoprim/sulfamethoxazole and meropenem. The MDR strains of *K. pneumoniae *were classified as organisms showing resistance to at least three classes of antibiotics including ceftazidime [18]. Resistance to ceftazidime identified by Vitek was used as the initial screening test for the presence of ESBL which was confirmed by E-test (AB Biodisk, Solna, Sweden) and double-disc synergy test which were performed according to the manufacturer's instructions and CLSI guidelines [17], respectively. A positive double disc synergy test was defined as enhancement of the zones of inhibition for ceftazidime and cefotaxime in the presence of clavulanic acid.

The MDR ESBL producing *K. pneumoniae *strains were assigned antibiotypes based on their resistance patterns.

### Pulsed Field Gel Electrophoresis

Pulsed-field gel electrophoresis (PFGE) was used to determine the relatedness of the ESBL producing strains of *K. pneumoniae*. The PFGE was performed as described previously with modifications [19]. Electrophoresis was carried out in 0.5 × TBE buffer using the Chef Mapper XA pulsed field electrophoresis system (Biorad, Hercules, California, U.S.A.). The conditions were 6 V/cm for 21 h at 12°C, with the pulse time ramped linearly from 1 s to 40 s. The molecular size marker included for comparison was *Saccharomyces cerevisiae *(Biorad, Hercules, California, U.S.A.). Following electrophoresis the gels were stained with ethidium bromide and photographed under ultraviolet light. The banding patterns were compared based on the criteria described by Tenover et al [20]. Isolates were considered indistinguishable if their restriction patterns had the same number of corresponding bands of the same apparent size and closely related for differences of 3 bands. Isolates which differed by 4 or more bands were considered unrelated.

The study was approved by the Ethics Committee in the Faculty of Medical Sciences of the University of the West Indies, Mona.

## Authors' contributions

NAC carried out the microbiological and molecular studies and drafted the manuscript. KRG and MS conceived of the study, participated in its design and coordination. All authors read and approved the final manuscript.
